# Streptococcus Group G of Unknown Source as a Rare Cause of Endogenous Endophthalmitis

**DOI:** 10.7759/cureus.36359

**Published:** 2023-03-19

**Authors:** Noelle Osei-Tutu Kudayah, Dragos Aconstantinesei, Gilda-Rae Grell

**Affiliations:** 1 Clinical Education, Touro College of Osteopathic Medicine, New York, USA; 2 Internal Medicine, Brookdale University Hospital Medical Center, Brooklyn, USA

**Keywords:** high risk, immunosuppression, infection, bacteremia, streptococcus group g, endophthalmitis

## Abstract

Endogenous endophthalmitis occurs in the setting of an intraocular infection secondary to the spread of bacterial, viral, or fungal pathogens with a high likelihood of permanent vision loss if left untreated. This case describes a 33-year-old male with numerous risk factors presenting with sudden onset of symptoms consistent with endogenous endophthalmitis secondary to *Streptococcus beta-hemolyticus *group G bacteremia of unknown source. He was treated with broad-spectrum antibiotics and analgesics with improvement in symptoms.

## Introduction

Endogenous endophthalmitis is defined as a severe intraocular infection affecting the vitreous and/or aqueous humors resulting from hematogenous spread of microorganisms [1]. It is considered a life-threatening medical emergency with the potential of vision loss. This condition is relatively rare in the United States and is typically secondary to bacteremia from sources including urinary tract infections, endocarditis, meningitis, or indwelling catheters. The most commonly involved bacterial microorganisms are Streptococci (*S. pneumoniae, S. milleri,* group A, group B), *Staphylococcus aureus (S. aureus),* and least commonly gram-negative bacilli. The most common fungal and mold species are *Candida albicans *and* Aspergillus flavus,* respectively [1]. Treatment modalities include prompt initiation of antibiotics, pain relievers, imaging, laboratory studies, and surgical management as needed [2]. 

## Case presentation

The patient is a 33-year-old African-American male with a past medical history of inflammatory arthritis, synovitis, acne, pustulosis, hyperostosis, osteitis (SAPHO) syndrome, lymphoma in remission post-adalimumab use, well-controlled hypertension, hyperlipidemia, and gastroesophageal reflux disease. His past surgical history includes a bilateral total hip replacement. He is currently taking adalimumab, prednisone, and methotrexate for the inflammatory arthritis and SAPHO syndrome, amlodipine-atorvastatin for hypertension and hyperlipidemia, and gastroesophageal reflux disease (GERD) is controlled with lifestyle modifications. His family history is non-contributory. 

He presented to the emergency room with sudden-onset fever, chills, headache, right eye pain, swelling, discharge, and decreased vision for the prior few hours. He endorsed that bright lights made symptoms worse, but had not taken any medications to alleviate the symptoms. The pain was described as severe, constant, sharp and radiated to the right side of forehead and scalp. He denied trauma, nausea, vomiting, loss of consciousness, and history of previous symptoms. 

**Figure 1:**
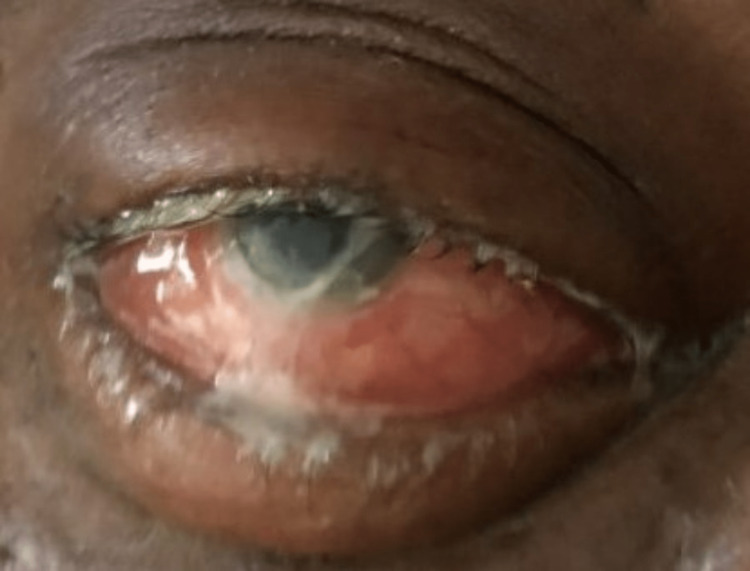
Right eye demonstrating severe swelling, proptosis, conjunctival erythema, and serous, white discharge

On initial presentation, his vital signs included a temperature of 98.5℉, heart rate of 94 beats per minute, respiratory rate of 20 breaths per minute, blood pressure of 153/99 mmHg, and a pulse oximetry of 100% on room air. Physical examination revealed an overweight male who appeared his stated age and was alert, oriented, and in mild acute distress. He was conversational, able to follow commands, and displayed normal mental status. Eye examination showed right eye proptosis, conjunctival erythema, serous discharge, swelling, and severe tenderness on palpation (Figure 1). Visual acuity completed using the Snellen's chart in the right eye was 20/40 and in the left eye 20/20. The left eye was normal, with no abnormalities noted. Remainder of head, ears, eye, nose, and throat (HEENT) exam revealed an atraumatic, normocephalic head, tympanic membranes with normal light reflex and no erythema, nares with pink mucosa and no discharge, and an oropharynx with moist mucous membranes, no erythema or exudates, and no lesions. Cardiovascular examination demonstrated regular rate and rhythm, normal S1, S2 with no thrills, heaves, rubs, or gallops. Lungs were clear to auscultation bilaterally with no wheezes, crackles, or rales. His abdomen was nondistended, nontender to palpation, no hepatosplenomegaly or masses present. On musculoskeletal and extremity exam, muscle strength was 5/5 bilaterally on upper and lower extremities, no edema noted, and tone and reflexes within normal limits. 

The patient was admitted and empirically started on intravitreal vancomycin, intravenous ceftazidime, intravenous dexamethasone and analgesics including morphine, hydromorphone, and high-dose acetaminophen. Two sets of blood cultures and vitreous fluid samples were collected on admission and the blood cultures identified the causative agent as *Streptococcus beta-hemolyticus* group G. Two days later, another set of samples was collected and showed no growth. The infectious disease consult team recommended that the original antibiotics be continued with the addition of intravenous ampicillin 2 grams every six hours for a duration of seven days for the patient’s bacteremia. It was also suggested that moxifloxacin, prednisolone, brimonidine, dorzolamide, and timolol eye drops be added to the current regimen and continued after discharge. Furthermore, daily ophthalmology evaluations and eye irrigation were advised.

**Figure 2:**
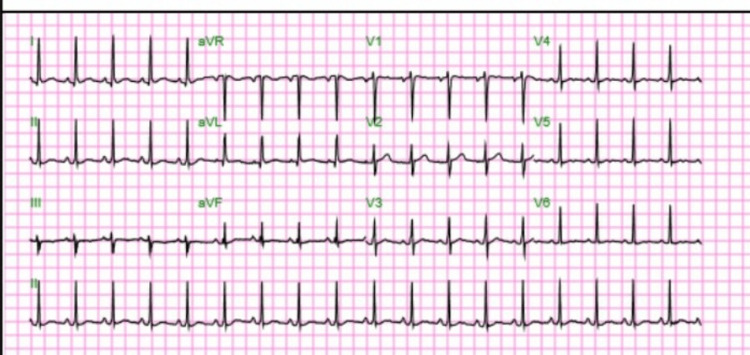
Electrocardiogram done on initial presentation demonstrating sinus tachycardia

After beginning treatment, imaging was ordered to identify a potential source of the bacteremia leading to endogenous endophthalmitis. An electrocardiogram ordered in the emergency department showed sinus tachycardia, but was otherwise within normal limits (Figure 2). A CT head and orbits was unremarkable and negative for any intracranial pathologies. A chest x-ray was ordered to rule out pneumonia as a source of infection and was also unremarkable for any cardiopulmonary processes. An echocardiogram revealed a normal left ventricle ejection fraction of 60-65% with mild tricuspid regurgitation, but no evidence of vegetations, thus ruling out infective endocarditis.

The patient continued the antibiotics for seven days in addition to high-dose pain medications as needed and noted mild improvement in symptoms with regard to pain. He noted that he was still experiencing vision loss in the right eye and was subsequently referred to an outpatient ophthalmology team that recommended a penetrating keratoplasty to be done in the coming weeks for full symptom relief. 

## Discussion

Endogenous endophthalmitis is a potentially life-threatening cause of permanent vision loss caused by hematogenous spread of bacterial, fungal, or viral infections. While the Streptococci species are one of the most common bacterial microorganisms involved in the disease course, group G has been noted to be a rare cause. *Streptococcus beta-hemolyticus *group G most commonly leads to septicemia, skin and soft tissue infections, septic arthritis, and endocarditis among many other manifestations [3]. When this microorganism is identified in the blood, especially in at-risk immunosuppressed patients, the likelihood of developing this disease significantly increases. 

Endogenous endophthalmitis is relatively rare and also has no preference based on age, race, or gender. In patients who are immunosuppressed, whether by medications or medical conditions such as those taking corticosteroids or those with diabetes, chronic kidney disease, HIV/AIDS, lymphoma, or another malignancy, the incidence of this disease is much higher [4]. The immunosuppressed state predisposes these patients to bacteremia which can spread through the blood to the highly vascularized ocular system [5]. 

The American Academy of Ophthalmology (AAO) notes that rapid diagnosis, treatment, and identification of the source of infection are crucial in preventing blindness and severe sepsis which may lead to poor prognostic outcomes [6]. A correct diagnosis stems from a good understanding of the pathophysiology of the disease and as well as a thorough history and examination, diagnostic tests, laboratory studies including blood and vitreous fluid samples, and imaging as needed [7]. Treatment must target the causative organism and source if one is identified and should include hospitalization, intravitreal antibiotic therapy, systemic intravenous antibiotic therapy, analgesics, and consults to appropriate departments as needed [8]. 

## Conclusions

Endogenous endophthalmitis is an intraocular infection that can have debilitating effects on vision, therefore early recognition, diagnosis, and treatment are vital. Initial treatment consists of broad-spectrum antibiotics including fluoroquinolones, aminoglycosides, cephalosporins, and clindamycin and is narrowed down based on microbiological identification and susceptibilities. The diagnostic workup must also include ocular fluid cultures, blood cultures, and imaging as needed. Bacterial infection with an identifiable source remains the most common cause of endogenous endophthalmitis with risk factors including underlying immunosuppression compounding to its increased incidence in these individuals. The prognosis is generally poor if diagnosed in its late stages and can ultimately lead to surgical intervention on the affected eye.
